# Near Real-Time Calving Detection in Grazing Cows Using GNSS-Derived Behavioral Anomalies

**DOI:** 10.3390/ani16142127

**Published:** 2026-07-09

**Authors:** Manuel J. García García, Eseró Padrón Tejera, María del Pilar Torralbo Muñoz, Dolores C. Pérez Marín, Mark G. Trotter, Anita Z. Chang, Justin Macor, Francisco Maroto Molina

**Affiliations:** 1 ISAG Research Group, Department of Animal Production, Universidad de Córdoba, Campus de Rabanales, Ctra. Madrid-Cádiz, km 396, 14071 Cordoba, Spain; g42gagam@uco.es (M.J.G.G.); esero.padron@uco.es (E.P.T.); g92tomum@uco.es (M.d.P.T.M.); dcperez@uco.es (D.C.P.M.); 2 Institute of Future Farming Systems, School of Health, Medical and Applied Science, Central Queensland University, Rockhampton, QLD 4701, Australia; m.trotter@cqu.edu.au (M.G.T.); a.chang@cqu.edu.au (A.Z.C.); j.macor@cqu.edu.au (J.M.)

**Keywords:** animal behavior, beef cattle, GPS tracking, grassland, parturition, precision livestock farming, rangeland

## Abstract

Timely calving detection is important but difficult in grazing systems because cows may calve in remote areas. This study developed and evaluated a near real-time calving alert system based exclusively on behavioral anomalies calculated from Global Navigation Satellite System (GNSS) collar data. Data were collected from 149 cows across three grazing farms in Spain and Australia, including 76 calving events. Individual, social, and herd-relative behavioral indicators were calculated every 30 min using 24 h rolling windows. Anomalies were defined as directional deviations from recent behavior using delta-self and Z-score transformations and were evaluated either at each 30 min timestamp or accumulated over rolling 3 h windows. Detection performance was assessed within the 20 days before calving, using a 48 h calving window as the reference period. Increasing the number of anomalies required to trigger a calving alert improved precision but reduced recall, while 3 h anomaly accumulation improved the stability of the alert system. Intermediate anomaly thresholds maximized F1-score, reaching 0.41 with individual behavioral indicators and 0.58 with the complete indicator set, irrespective of whether anomalies were evaluated at 30 min intervals or accumulated over 3 h. Alerts and indicator-specific anomalies were concentrated during the final two days before calving, mainly reflecting increased spatial separation and reduced movement relative to the group. These results support GNSS-based anomaly accumulation as a transparent and flexible approach for calving detection under grazing conditions.

## Simple Summary

Detecting when cows are about to calve is important because farmers may need to intervene if there are problems during birth or if the newborn calf needs help. This is especially difficult in grazing systems, where cows can calve in large and remote paddocks, or other areas that are not easy to inspect. This study tested whether tracking collars, which record the location of cows, can be used to detect changes in behavior before calving. We monitored cows on three grazing farms in Spain and Australia and analyzed changes in their movement and position relative to other cows. The system generated alerts when several unusual behavioral changes occurred within a short period. Alerts were more common during the two days before calving, especially when cows moved away from the herd or reduced their locomotion compared with the rest of the group. These results suggest that location data from tracking collars can help detect cows approaching calving without the need for additional sensors. This approach could help farmers locate cows due to calve more efficiently, improve supervision during the calving season, and support animal welfare in extensive grazing systems.

## 1. Introduction

Timely detection of calving is important for improving reproductive management, animal welfare, and calf survival in beef cattle systems. Calving is a critical event because delayed or missed identification can reduce the opportunity for intervention in cases of dystocia, postpartum complications, or neonatal weakness, and may contribute to calf mortality and reduced reproductive efficiency [1,2]. Early identification of cows approaching calving can also support better allocation of labor during the calving season, improving herd management efficiency [3,4].

Monitoring calving is particularly challenging in extensive and semi-extensive grazing systems. In housed systems, cows can be inspected frequently or monitored using cameras and other sensors. For example, Rutten et al. [5] used images recorded in a dairy barn at 5 min intervals as the gold standard for determining calving onset when evaluating an accelerometer ear tag, illustrating the high temporal resolution that can be achieved under controlled housing conditions. In contrast, continuous observation is often neither physically nor economically feasible in extensive rangelands, where cows may calve in remote paddocks, woodland, or areas with limited visibility or difficult access [6]. As a result, the exact time of birth may remain unknown, and calving is commonly recorded only when the cow is first observed with the calf.

The practical importance of this monitoring limitation is reinforced by the concentration of calf losses around the time of birth. In range beef cattle, Patterson et al. [7] reported that 57.4% of calf deaths occurred within the first 24 h after birth and that dystocia was the main category of calf loss during the first 96 h. Similarly, a study on extensive Australian beef systems reported an average pre-weaning calf mortality of 9.5%, with 67% of deaths occurring during the first week of life [8]. Comparable losses have also been reported in European grazing beef systems. Santos et al. [9] observed a pre-weaning calf mortality of 5.7% in free-range farms in southern Portugal, whereas Norquay et al. [10] reported a mean perinatal mortality of 5.1% across 23 beef herds in Scotland, with values ranging from 1.6% to 12.4% among herds. In extensively managed German Angus suckler herds, Hohnholz et al. [11] reported a dystocia prevalence of 3.4% and a perinatal mortality rate of 4.3%. These studies demonstrate that clinically relevant losses occur around parturition under extensive management and that delayed detection may reduce opportunities for timely supervision or intervention during the period of greatest risk. This creates a need for autonomous monitoring tools capable of detecting behavioral changes associated with calving under real farming conditions [12].

Sensor-based technologies offer new opportunities for remote reproductive monitoring. Among them, Global Navigation Satellite System (GNSS) trackers are especially relevant for grazing cattle because they provide information on animal location, enabling the study of movement trajectories, space use, and social position within the herd [13,14]. These data can be used to infer behavioral changes associated with calving, including reduced walking distance, altered movement patterns, and increased separation from herd mates [15,16,17].

Previous studies have shown that GNSS-based indicators can capture behavioral changes around parturition in grazing ruminants. In beef cattle, García-García et al. [11] developed a GNSS-based framework using trajectory and herd-relative metrics, including distance traveled, path sinuosity, distance to the herd centroid, and distance to the nearest neighbor, and reported consistent changes during the 24–48 h preceding calving. In sheep, Fogarty et al. [18] used GNSS data to detect lambing-related changes in movement and social behavior under grazing conditions. Similar spatial responses around parturition have been reported in mouflon [19,20] and bighorn sheep [21,22]. Collectively, these studies support the use of movement and social-behavior indicators as non-invasive proxies for detecting parturition in grazing animals.

Recent research has also explored more complex sensor-based approaches for parturition detection. Fogarty et al. [23] integrated GNSS, accelerometer, and weather data for lambing detection, while Chang et al. [24] combined GNSS, accelerometer-based rumination, walk-over weight, and weather data to detect prepartum states in cattle. More recently, Wang et al. [25] also developed a calving detection approach for rangeland conditions using GNSS and accelerometer data. These studies demonstrated the potential of multi-sensor systems for parturition detection, but their deployment under extensive grazing conditions may involve some operational trade-offs. Combining GNSS with accelerometers or other sensors increases device cost, the number of hardware components that may require maintenance, and the volume and frequency of data streams that must be acquired, transmitted, synchronized, and processed. Some sensors may also require stable orientation, periodic calibration, or sensor-specific validation to ensure consistent interpretation of the recorded signals. For example, Wang et al. [25] configured collar-mounted devices to transmit GNSS locations every 60 min but accelerometer-derived motion information every 2 min. Their system required three stationary LoRaWAN base stations and one mobile base station to provide coverage and minimize packet collision and data loss across the study area. In addition, a 500 g counterweight was fitted to each collar to maintain the device in an upright orientation and optimize data acquisition and transmission. Although this configuration achieved an estimated battery life exceeding one year, it illustrates the additional hardware design, communication infrastructure, and data-integration requirements that may accompany even relatively simple multi-sensor platforms. These requirements may increase deployment and maintenance costs and become increasingly relevant as herd size and the number of connected devices grow. All sensor systems used under grazing conditions must also withstand dust, rain, impacts, and prolonged outdoor exposure; however, integrating several sensing components may further increase device complexity and the number of potential failure points. A GNSS-only approach avoids the need to acquire and integrate a separate high-frequency sensor stream and can therefore reduce system complexity, data-transmission demands, and calibration requirements, although it remains subject to the usual limitations of GNSS trackers. Therefore, there is value in evaluating whether behavioral changes associated with calving can be detected using GNSS information alone, particularly when commercial tracking collars are already deployed in many commercial farms for other grazing-management purposes.

Previous studies have provided valuable post hoc descriptions of peripartum behavior, often based on hourly or daily summaries of behavioral data [16,17]. However, these approaches were primarily designed to characterize behavioral changes associated with calving rather than to generate operational near real-time alerts. Consequently, an important research gap remains in the development of early-warning frameworks that can process incoming data at short intervals and support practical deployment under commercial field conditions. A further limitation is that the studies addressing detection approaches mostly rely on supervised learning. Supervised models require accurately labeled events for training and validation, but in grazing systems the exact time of calving is rarely known. Assigning a precise timestamp for calving, which is usually only confirmed retrospectively, may introduce false precision. Therefore, there is a need for methods that can operate under weakly labeled field conditions, where the event is known to have occurred within a time window, but its exact timing remains uncertain.

Anomaly detection is particularly suitable for calving monitoring because prepartum behavior is unlikely to follow an identical pattern across cows. Instead, calving-related changes are more plausibly expressed as short-term but sustained deviations from each animal’s recent behavioral baseline, and the magnitude of these deviations may also vary among individuals, farms, and management conditions. Therefore, the relevant signal is not necessarily a uniform herd-wide response, but an unusual change in the behavior of a particular cow, interpreted relative to both her own recent activity and, where available, the concurrent behavior of her groupmates.

Anomaly detection methods are also well suited to weakly labeled datasets, which are common in many real-world applications [26]. Under commercial grazing conditions, calving occurrence can often be assigned only to a broader observation period rather than to an exact timestamp. In time-series data, anomalies may correspond not only to isolated observations but also to subsequences that deviate from expected temporal behavior. Sliding windows can therefore be used to compare incoming observations with recent or predicted patterns [27]. This is directly relevant to calving detection, where a single behavioral deviation may reflect normal grazing variability, environmental effects, management events, or sensor error, whereas the accumulation of several biologically meaningful deviations within a short period may indicate a sustained behavioral shift compatible with the peripartum period.

The present study aims to address these gaps by proposing a flexible, anomaly-based calving detection framework relying exclusively on GNSS tracking data from commercially available collars. Unlike approaches that require exact event labels or multiple sensor platforms, the proposed method is based on the identification of deviations in GNSS-based behavioral indicators relative to each cow’s recent behavior and to the behavior of its groupmates. A further goal of the study was to evaluate how detection performance is affected by the indicator set, the number of anomalies, and their short-term temporal accumulation.

## 2. Materials and Methods

### 2.1. Study Locations

The study was conducted on three beef cattle farms: two in Spain and one in Australia. The Spanish farms were ‘Fuente del Perro’ (FDP) in Pedroche, Córdoba, and ‘El Ahijón’ (EAH) in Zorita, Cáceres. The Australian farm, known as ‘Belmont Station’ (BES), was located near Rockhampton, Queensland.

‘Fuente del Perro’ and ‘El Ahijón’ are commercial farms located within the Spanish dehesa, an agrosilvopastoral ecosystem characterized by scattered trees, mainly *Quercus* spp., shrublands, and natural pastures. Both sites have a Mediterranean climate according to the Köppen classification (Csa), with average annual rainfall of approximately 400–600 mm, mainly concentrated in autumn and spring. Average daily temperatures typically range from around 2 °C in winter to more than 33 °C in summer. ‘Fuente del Perro’ covers 208 ha and is subdivided into 14 grazing paddocks ranging from 3.3 to 28.8 ha. ‘El Ahijón’ covers 260 ha and is divided into eight paddocks ranging from 9.4 to 72.6 ha. At both sites, paddocks are enclosed using a combination of stone walls and wire fencing.

‘Belmont Station’ is an experimental farm located in Central Queensland. It has a dry tropical climate according to the Köppen classification (Aw). Summers are hot and humid, with maximum temperatures frequently exceeding 35 °C, whereas winters are dry, with minimum temperatures around 10 °C. Mean annual rainfall is approximately 800–1000 mm, mostly occurring between December and March. The farm covers approximately 3440 ha and consists mainly of open grasslands interspersed with *Eucalyptus* spp. trees. The grazing area is divided into 90 paddocks by wooden and wire fences.

### 2.2. Cattle Monitoring

#### 2.2.1. ‘Fuente Del Perro’ Farm

The herd at FDP consisted of 75 adult cattle, including two bulls and 73 Limousin and crossbred suckler cows aged between 3 and 13 years. Animals were moved between paddocks every several weeks according to forage availability and farm management requirements. A total of 57 females, representing 76% of the herd, were randomly selected and fitted with commercial GNSS collars (Digitanimal Ltd., Madrid, Spain) from August 2019 to April 2020. The collars recorded animal location every 30 min and transmitted the data to a cloud server using Sigfox connectivity. To ensure adequate data transmission, a dedicated Sigfox antenna was installed on the farm.

Calving was monitored through twice-daily visual inspections by farm staff. A calving event was recorded when a newborn calf was observed with the cow or when the cow showed clear signs of parturition, such as udder distension, abdominal hollowing, relaxation of the sacrosciatic ligaments, or the presence of a visible amniotic sac, even if the calf was not yet visible.

#### 2.2.2. ‘El Ahijón’ Farm

The herd at EAH comprised 94 adult cows, including Limousin and crossbred animals aged between 3 and 14 years. A total of 20 commercial GNSS collars (Digitanimal Ltd., Madrid, Spain) were available, which were rotated among 42 cows, representing 45% of the herd, over an eight-month period, from November 2023 to April 2024 and from August to September 2024. Collar allocation followed the farm’s routine reproductive-management strategy. At the beginning of each monitoring period, collars were fitted to cows that the farmer considered most likely to calve in the near term, based on expected calving dates and visual assessment. After a monitored cow calved, or when subsequent observation indicated that calving was not imminent, the collar was transferred to another cow with an estimated calving date approaching. Therefore, monitored cows were not randomly selected and should not be considered fully representative of the entire herd. Rather, this dataset reflects a targeted monitoring strategy aimed at maximizing the number of impending calvings that can be covered when collar availability is limited. The collars recorded location data at 30 min intervals and transmitted it to the cloud server via GSM connectivity.

Grazing management and calving monitoring were conducted as described for FDP, with cows moved between paddocks according to pasture availability and calving events identified through twice-daily visual inspections based on either direct calf observation or clear signs of parturition.

#### 2.2.3. ‘Belmont Station’ Farm

At BES, the experimental herd comprised 50 three-year-old heifers, predominantly tropical composite cattle. All animals (100%) were fitted with i-gotU GNSS collars (Mobile Action Technology Inc., New Taipei City, Taiwan) configured to record location data at approximately 5 min intervals from September to November 2022. Location data were stored on board and downloaded after collar retrieval.

Animals were managed under extensive grazing conditions, with paddock allocation determined by seasonal forage availability. Calving was monitored through twice-daily visual observations conducted by farm staff and/or researchers. A calving event was recorded when a newborn calf was observed with the dam or when clear signs of parturition were detected.

The main characteristics of herds, monitoring systems, and study periods at the three farms are summarized in Table 1.

The three farms included in the study represent contrasting and practically relevant GNSS-collar deployment scenarios. At BES, all animals were monitored, representing a favorable situation in which full-herd coverage was available. At FDP, collar availability was slightly lower than herd size, but the same animals remained monitored throughout the study. In contrast, EAH represented a more resource-constrained scenario that may be common on commercial farms, where a limited number of collars is rotated among cows considered most likely to calve in the near term. This design allowed the proposed framework to be evaluated under different levels of collar availability and monitoring intensity, ranging from complete herd coverage to targeted collar allocation.

### 2.3. GNSS Data Preprocessing

Raw GNSS data retrieved from the collars were processed using a standardized workflow designed to improve spatial accuracy, temporal consistency, and comparability across farms. Data management and calculations were performed using R version 4.5.0 [28]. Because the three datasets differed in collar model, communication system, and sampling frequency, preprocessing focused particularly on reducing differences associated with data completeness and temporal resolution.

First, daily records were screened for data completeness. Cow-days were retained only when at least 50% of the possible GNSS fixes were available for each sampling interval, and no data gap exceeded 6 consecutive hours. Accordingly, the minimum was 24 valid fixes for the Spanish farms, with 48 fixes expected at 30 min intervals, and 144 valid fixes per day for BES, with 288 fixes expected at 5 min intervals. These criteria were used to exclude trajectories with insufficient temporal coverage and to limit the influence of differences in data transmission and storage among collar systems.

Then, to harmonize data from collars with different sampling intervals, all retained trajectories were resampled to exact 30 min timestamps through linear interpolation between adjacent GNSS fixes using the R package *trajr* 1.5.1 [29]. This temporal resolution matched the sampling interval of the Spanish datasets, allowed behavioral indicators to be calculated consistently across farms, and represented a realistic compromise between temporal detail, battery life, and data-transmission requirements in commercially deployable grazing-monitoring systems.

The procedure also generated interpolated positions at missing expected timestamps within otherwise retained trajectories. Although this step reduced the differences associated with temporal resolution and missing-data patterns, resampling may smooth fine-scale movement and affect the values of some behavioral indicators. In addition, potential device-specific differences in GNSS positional accuracy could not be fully removed and were therefore considered when interpreting farm-specific results.

### 2.4. Calculation of Behavioral Indicators

Behavioral indicators used in this study were adapted from those described by García-García et al. [17]. The final set of indicators was selected because they have been shown to capture changes in movement, spatial use, and social isolation associated with the peripartum period. Two types of indicators were calculated. Individual indicators described the movement pattern of each cow independently, including total trajectory length, number of trajectory segments exceeding 25 m, and trajectory sinuosity. Social and herd-relative indicators described the behavior of each cow in relation to its groupmates and included distance to the nearest neighbor, relative trajectory length, relative distance (start-to-end displacement), relative number of segments exceeding 25 m, relative maximum segment length, relative straightness, relative sinuosity, relative distance to the nearest neighbor, relative mean distance to herd centroid, and relative maximum distance to herd centroid. A summary of the behavioral indicators is provided in Table 2.

All behavioral indicators were calculated at each 30 min timestamp using a rolling 24 h window, corresponding to the current position and the previous 48 resampled positions. This resulted in 49 positions per animal and time interval.

On the commercial farms (FDP and EAH), cows were dynamically regrouped into different grazing paddocks based on their nutritional and reproductive status. Therefore, for the calculation of social and herd-relative indicators, cows were automatically assigned to paddock-based groups. Each GNSS position was spatially matched to the corresponding grazing paddock using paddock boundary shapefiles created in QGIS version 3.16.13 [30]. A group was defined as the set of monitored cows occupying the same paddock during the same period, and cows were reassigned whenever they moved to a different paddock. Paddock occupancy was used as a management-based spatial constraint to prevent animals located in different enclosures from being included in the same social calculations, rather than to assume that all cows within a paddock formed a permanently cohesive social unit.

Only GNSS positions from individuals within the same group were used to calculate social and herd-relative indicators. Within these groups, the indicators captured social structure at different spatial scales. Distance to the herd centroid summarized each cow’s position relative to the monitored group as a whole, whereas distance to the nearest monitored neighbor reflected more local social proximity and was less dependent on the assumption of a single cohesive group.

**Table 2:** Individual, social and herd-relative behavioral indicators associated with the peripartum period.

Acronym	Description (Unit)
Individual indicators	
leng	Length of the trajectory traveled by each animal (m)
n25	Number of trajectory segments (straight lines between consecutive fixes) longer than 25 m
sin	Estimate of the tortuosity of a path. It is a function of both the mean cosine of turning angles and the length of trajectory segments, ranging from 0 (straight path) to 1 (very curved path). It was calculated according to [31]
Social and herd-relative ^1^ indicators	
dist_near	Average of the straight-line distance from each location fix of each animal to the location fix of the nearest groupmate (m)
r_leng	Length of the trajectory traveled by each animal, relative to the average trajectory length of its groupmates (%)
r_dist	Straight-line distance between the start and the end of the trajectory of each animal, relative to the average value of this distance for its groupmates (%)
r_n25	Number of trajectory segments above 25 m long, relative to the average number of segments above 25 m long for its groupmates (%)
r_max_step	Maximum length of trajectory segments, relative to the average maximum length of segments for its groupmates (%)
r_str	Straightness index (trajectory distance divided by trajectory length) of the trajectory of each animal, relative to the average straightness of its groupmates’ trajectories (%)
r_sin	Tortuosity of the trajectory of each animal, relative to the average tortuosity of its groupmates’ trajectories (%)
r_dist_near	Average of the straight-line distance from each location fix of each animal to the location fix of the nearest groupmate, relative to the average distance to nearest animal for its groupmates (%)
r_mean_dist_cen	Average of the straight-line distance from each location fix of each animal to herd centroid, relative to the average distance to herd centroid for its groupmates (%)
r_max_dist_cen	Maximum of the straight-line distance from each location fix of each animal to herd centroid, relative to the average maximum distance to herd centroid for its groupmates (%)

^1^ Herd-relative indicators were calculated as ${indicator}_{i}/\left(\sum_{1}^{n}{indicator}_{j}/n\right)-1$, where *i* = calving cow, *j* = each groupmate, *n* = group size.

### 2.5. Anomaly Detection

Behavioral anomalies were detected using a two-step transformation applied independently to each behavioral indicator. First, a delta-self transformation was used to quantify deviations from each cow’s recent behavior. For each indicator, the relative change at time *t* was calculated as:

(1) $${∆I}_{t}=\frac{I_{t}-\bar{I_{t-24h}}}{\bar{I_{t-24h}}}$$

where $I_{t}$ is the value of the indicator at time *t*, and $\bar{I_{t-24h}}$ is the mean value of the same indicator for the same cow during the preceding 24 h period. This transformation allowed each observation to be interpreted relative to the animal’s own recent behavioral baseline. A 24 h reference window was selected to capture a complete circadian cycle of cattle behavior. Locomotion, feeding, rumination, and resting activity vary systematically according to the time of day, and changes in the circadian rhythmicity of cattle have been associated with reproductive and health events, as well as with environmental conditions [32]. Using a shorter reference period could result in the current observation being compared predominantly with a different phase of the daily activity cycle. Conversely, a longer window would provide a smoother baseline but could reduce sensitivity to short-term behavioral changes preceding calving. The 24 h window was therefore selected a priori as a biologically informed compromise between accounting for circadian variation and retaining sensitivity to recent behavioral deviations.

Second, delta-self values were standardized within each cow using a Z-score transformation to make deviations comparable across indicators and time intervals:

(2) $$Z_{t}=\frac{{∆I}_{t}-\mu_{∆I}}{\sigma_{∆I}}$$

where ${∆I}_{t}$ is the delta-self value at time *t*, and $\mu_{∆I}$ and $\sigma_{∆I}$ are the mean and standard deviation of delta-self values for the corresponding indicator calculated within the same cow. This standardization allowed unusually large deviations to be identified relative to each animal’s own distribution of behavioral changes.

Inspired by Wang et al. [25], anomaly thresholds were defined separately for each behavioral indicator using the empirical distribution of its standardized values after excluding observations recorded during the 48 h period preceding calving. For indicators expected to decrease around calving, observations below the 1st percentile of their respective distributions were classified as anomalies. For indicators expected to increase, observations above the 99th percentile were classified as anomalies. This approach accounted for differences in the variability and distribution of each indicator while defining anomalies from observations not temporally associated with the immediate prepartum period.

Decreases were considered anomalous for total trajectory length, number of segments exceeding 25 m, relative trajectory length, relative number of segments exceeding 25 m, and relative maximum segment length, reflecting reduced locomotion around calving. Increases were considered anomalous for trajectory sinuosity, distance to the nearest neighbor, relative distance, relative sinuosity, relative distance to the nearest neighbor, and relative distance to herd centroid, reflecting altered movement patterns, social isolation, and spatial separation during the prepartum period.

To identify sustained behavioral disruption rather than isolated deviations, anomaly counts were accumulated over a rolling 3-h window, equivalent to six consecutive 30 min intervals. The rationale for this calculation was that isolated anomalies may reflect transient noise or short-term behavioral variation, whereas the concentration of multiple anomalies within a short period was considered more likely to represent a biologically meaningful disruption associated with calving.

### 2.6. Evaluation of the Calving Detection System

The performance of the anomaly-based calving detection system was evaluated by comparing generated alerts with confirmed calving events. Because the exact timing of parturition was unknown under extensive grazing conditions, a calving window was defined for each event as the 48 h period preceding the date and time at which the cow was first observed with the calf or showed clear signs of parturition. This window was used as the reference period for evaluating whether alerts were temporally associated with calving.

Alerts were generated when the number of directional behavioral anomalies reached or exceeded a given threshold. Rather than defining a single anomaly count threshold a priori, the effect of different thresholds on detection performance was evaluated. This allowed the sensitivity of the system to be assessed under different alerting criteria and provided a basis for identifying thresholds that balanced calving detection and false alert occurrence.

System performance was evaluated using a 2 × 2 analytical framework based on two factors: indicator set and temporal aggregation. The first factor compared alerts generated using only individual indicators, which can be calculated from a single GNSS collar, with alerts generated using the complete indicator set, which also included social and herd-relative indicators and therefore required multiple collared animals within the same group. The second factor compared anomaly counts calculated at each 30 min timestamp with anomaly counts accumulated over a rolling 3 h window. This resulted in four evaluation scenarios: individual indicators at 30 min resolution, individual indicators accumulated over 3 h, complete indicator set at 30 min resolution, and complete indicator set accumulated over 3 h. This design allowed us to assess both the added value of herd-relative information and the effect of temporal accumulation on calving detection performance.

Performance metrics were calculated within a 20-day evaluation period preceding each calving event, with day −1 defined as the 24 h before confirmed calving and day −20 as the first day included in the evaluation. This period was selected to represent a practical monitoring horizon for detecting prepartum behavioral changes and generating calving alerts. Therefore, classification metrics were not calculated over the entire monitoring period, but only over the 20 days preceding each confirmed calving event.

Within this 20-day evaluation period, performance metrics were calculated on a cow-day basis. For each cow, each day was classified as either within or outside the 48 h calving window and as either with or without a system-generated alert. Multiple alerts occurring within the same cow-day were therefore counted only once. The cow-day was selected as the evaluation unit because the exact timestamps of calving events were not available. Calving was identified through twice-daily visual inspection and could therefore only be assigned to a broader prepartum period. Evaluating performance at 30 min resolution would have introduced false temporal precision because intervals without alerts could not be reliably classified as false negatives when the interval closest to the true calving event was unknown. In addition, consecutive alerts from the same cow were likely to reflect a single sustained behavioral episode rather than independent detections. Collapsing repeated alerts within the same cow-day therefore reduced pseudo-replication and provided an evaluation scale consistent with the temporal resolution of the reference data. A cow-day within the calving window was classified as a true positive (*TP*) when one or more alerts were generated, and as a false negative (*FN*) when no alert was generated. Conversely, a cow-day outside the calving window was classified as a false positive (*FP*) when one or more alerts were generated and as a true negative (*TN*) when no alert was generated. This classification assessed whether the system generated a practically relevant warning during the calving window rather than the total number of alerts produced. Based on these outcomes, performance was assessed using precision, recall, and F1-score:

(3) $$Precision=\frac{TP}{TP+FP}$$

(4) $$Recall=\frac{TP}{TP+FN}$$

(5) $$F1\text{-}score=2\times \frac{Precision\times Recall}{Precision+Recall}$$

Precision quantified the proportion of alert-positive cow-days that occurred within the calving window, while recall quantified the proportion of calving-window cow-days correctly identified by the system. The F1-score was used as a combined measure of precision and recall. This evaluation framework allowed the effect of the anomaly threshold, indicator set, and temporal accumulation strategy to be quantified while accounting for the uncertainty associated with calving observations under grazing conditions.

## 3. Results

### 3.1. GNSS Data and Calving Records

Across the three farms, a total of 2,093,834 raw GNSS positions were retrieved from 149 monitored cows during the study periods. The number of raw GNSS positions differed among farms, with 717,330 positions recorded at FDP, 215,473 at EAH, and 1,161,031 at BES.

The application of the data-completeness criteria detailed in Section 2 resulted in the exclusion of 3.6% of cow-days at FDP, 3.1% at EAH, and 2.1% at BES. After data cleaning and resampling to a common 30 min interval, 1,143,224 positions were available for subsequent analysis (767,209 at FDP, 238,937 at EAH and 137,078 at BES). At ‘Belmont Station’, the reduction in records mainly reflected the resampling of the original 5 min data to 30 min intervals, rather than data exclusion alone.

A total of 76 calving events were recorded during the monitoring periods. Of these, 23 occurred at FDP, 18 at EAH, and 35 at BES. Eight events, one at EAH and seven at BES, were excluded from the study due to collar failure before parturition. Calving records were used to define the 48 h calving windows for evaluating the anomaly-based detection system.

### 3.2. Indicator-Specific Anomaly Thresholds

**Figure 1:**
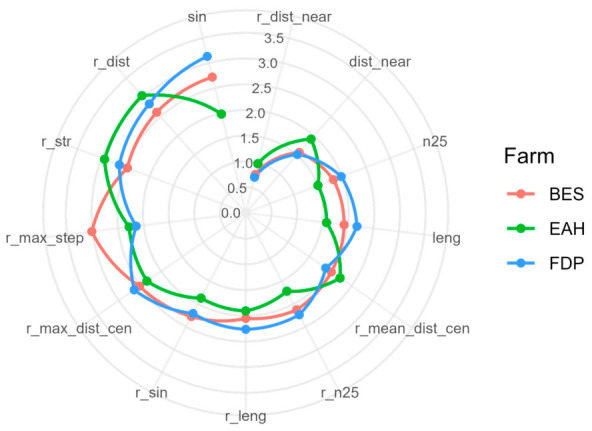
Indicator-specific anomaly thresholds corresponding to the 1st or 99th percentile depending on the biologically expected direction of change for each indicator around calving.

Indicator-specific anomaly thresholds varied substantially among behavioral metrics but showed broadly consistent patterns across farms (Figure 1). Indicators with comparatively low or high percentile-based thresholds tended to occupy similar relative positions at FDP, EAH, and BES, despite differences in collar technology, management, animal population, and monitoring conditions. This consistency indicates that the distributional characteristics of the standardized changes were primarily associated with the type of behavioral indicator rather than being farm-specific.

The upper-tail threshold for relative distance to the nearest neighbor was below 1 at all farms, whereas the corresponding threshold for trajectory sinuosity exceeded 2.5 in two of the three farms. Other indicators showed intermediate values, with generally similar rankings among farms.

The similarity of the threshold profiles among farms suggests that each indicator retained broadly comparable distributional behavior across contrasting production systems. Nevertheless, the absolute values were not identical, supporting the use of empirically derived thresholds (for each indicator and farm) rather than a single universal cut-off.

### 3.3. Detection Performance According to Anomaly Threshold

**Figure 2:**
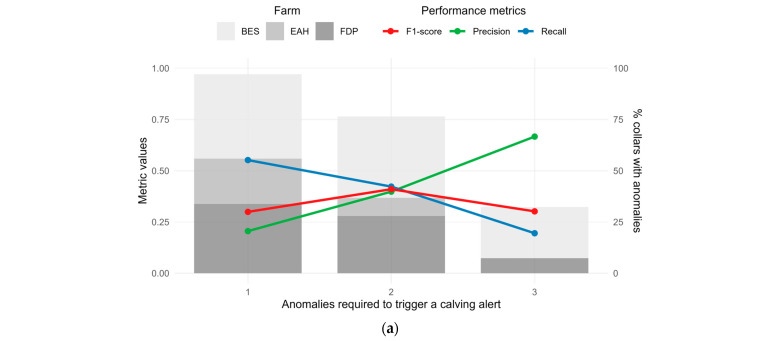

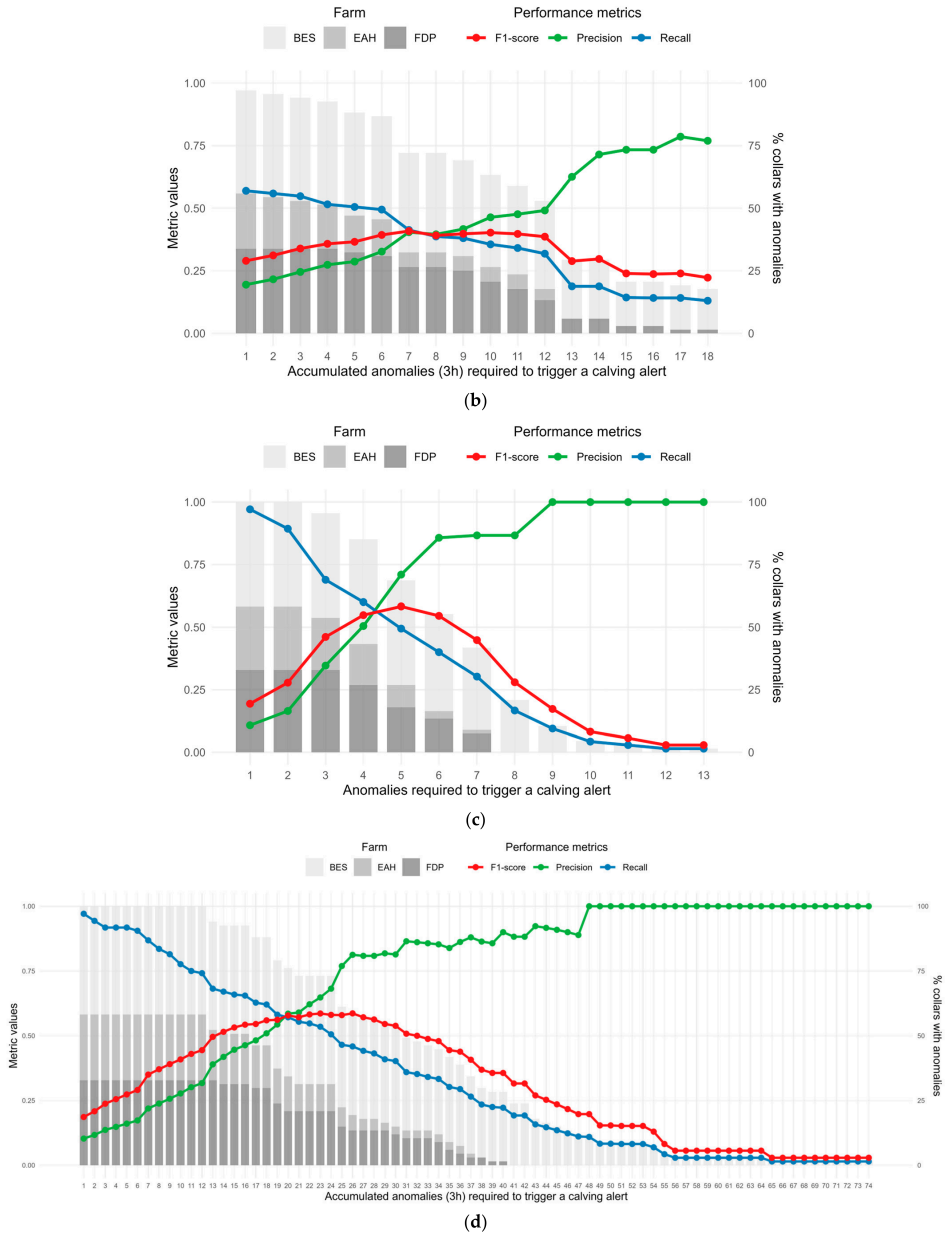
Calving detection performance metrics according to anomaly threshold: (**a**) anomalies at each 30 min timestamp calculated with a single collar; (**b**) anomalies accumulated over 3 h windows calculated with a single collar; (**c**) anomalies at each 30 min timestamp calculated with multiple collars; (**d**) anomalies accumulated over 3 h windows calculated with multiple collars.

The performance of the calving detection system varied markedly according to the number of anomalies required to trigger an alert and the configuration of the detection system (Figure 2a–d). The maximum possible number of anomalies differed among scenarios because of the number of indicators included and whether anomalies were evaluated at a single 30 min timestamp or accumulated over time. In the single-collar scenario, based only on individual indicators, a maximum of three anomalies could occur at each 30 min timestamp, whereas up to 18 anomalies could accumulate over a 3 h rolling window. In the multi-collar scenario, based on the complete set of 13 individual, social, and herd-relative indicators, the maximum possible number of anomalies was 13 at each 30 min timestamp and 78 over a 3 h rolling window. Therefore, anomaly thresholds should be interpreted relative to the maximum number of possible anomalies in each scenario.

When only individual indicators were evaluated at each 30 min timestamp, 97% of collars generated at least one alert when a single anomaly was sufficient to trigger an alert (Figure 2a). However, this proportion decreased sharply as the threshold increased, with 76% of collars reaching two anomalies and 30% reaching three anomalies. Farm-specific patterns were also evident. Collars from EAH did not generate alerts above the two-anomaly threshold, whereas three simultaneous anomalies were mostly observed in collars from BES. Precision increased from approximately 20% at one anomaly to 66% at three anomalies, but recall declined rapidly and was 19% at the strictest threshold. The maximum F1-score was 0.41, obtained at a threshold of two anomalies.

Accumulating individual-indicator anomalies over a 3 h rolling window produced a broader range of possible thresholds and a more gradual decline in the proportion of alert-positive collars (Figure 2b). Above 90% of collars generated up to four accumulated anomalies, and, as expected, the proportion of collars with alerts decreased progressively as the threshold increased. Alerts from EAH were concentrated at low and intermediate thresholds and disappeared above 13 accumulated anomalies, whereas BES collars remained represented across the full threshold range. ‘Fuente del Perro’ farm also contributed mainly at low and intermediate thresholds. Precision increased progressively with the number of accumulated anomalies, while recall decreased. The highest F1-scores, around 0.4, were observed at intermediate thresholds, between six and 12 accumulated anomalies, suggesting that temporal accumulation improved the ability to distinguish repeated behavioral deviations from isolated anomalies without requiring excessively strict alert criteria.

When the complete indicator set was evaluated at each 30 min timestamp, all collars generated at least one alert at the lowest threshold (Figure 2c). The proportion of GNSS collars producing alerts remained high at thresholds of two and three anomalies but declined markedly from four anomalies onwards. At higher thresholds, alerts were increasingly concentrated in fewer collars and farms. As in previous scenarios, EAH contributed mainly at the lowest thresholds and disappeared as the anomaly threshold increased, whereas BES remained the main contributor at the highest thresholds. Precision increased sharply with the anomaly threshold, reaching 100% when nine or more anomalies were required. Although precision reached 100% at these highly restrictive thresholds, this was associated with the detection of only a small number of calving events. Therefore, these thresholds should not be interpreted as the most useful operating points. The maximum F1-score was 0.58, obtained at a threshold of five anomalies. This indicates that requiring several but not most indicators to be anomalous provided the best balance between precision and recall.

The most stable pattern was observed when anomaly counts derived from the complete indicator set were accumulated over a 3 h rolling window (Figure 2d). In this scenario, all collars generated alerts at low thresholds (below 12 anomalies required to trigger an alert), and the proportion of alert-positive collars declined progressively as the number of accumulated anomalies increased. Alerts from the Spanish farms were mainly concentrated at low and intermediate thresholds, whereas BES collars continued to generate alerts at higher thresholds (exclusively over 40 accumulated anomalies). Precision increased steadily with the anomaly threshold and reached 100% at high thresholds (above 48 accumulated anomalies), while recall declined gradually as the threshold became increasingly restrictive. The F1-score reached its highest values, around 0.58, at intermediate thresholds, approximately between 22 and 26 accumulated anomalies. This range provided the best compromise between reducing false positives and maintaining detection of calving-window cow-days.

Overall, increasing the anomaly threshold consistently improved precision but reduced recall. The proportion of collars that generated calving alerts also decreased with increasing thresholds, showing that stricter criteria restricted detection to fewer animals and, in most scenarios, mainly to BES cows. Temporal accumulation over 3 h improved the stability of the detection system, particularly when the complete set of indicators was used. These results support the use of intermediate anomaly thresholds, because isolated anomalies were frequent across collars, whereas the accumulation of multiple anomalies within a short period was more specifically associated with calving-related behavioral disruption.

### 3.4. Temporal Distribution of Alerts

**Figure 3:**
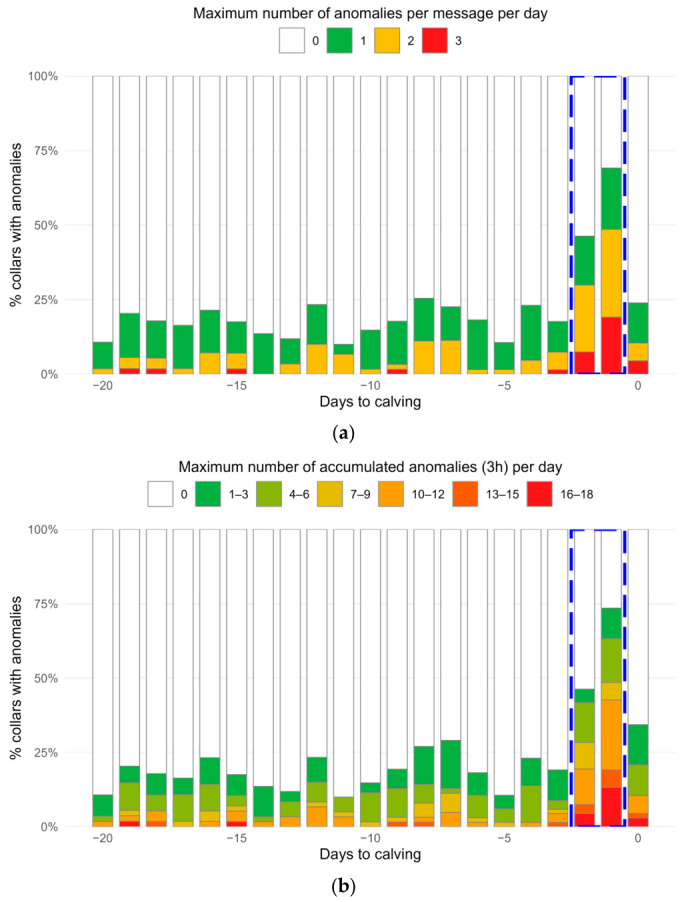

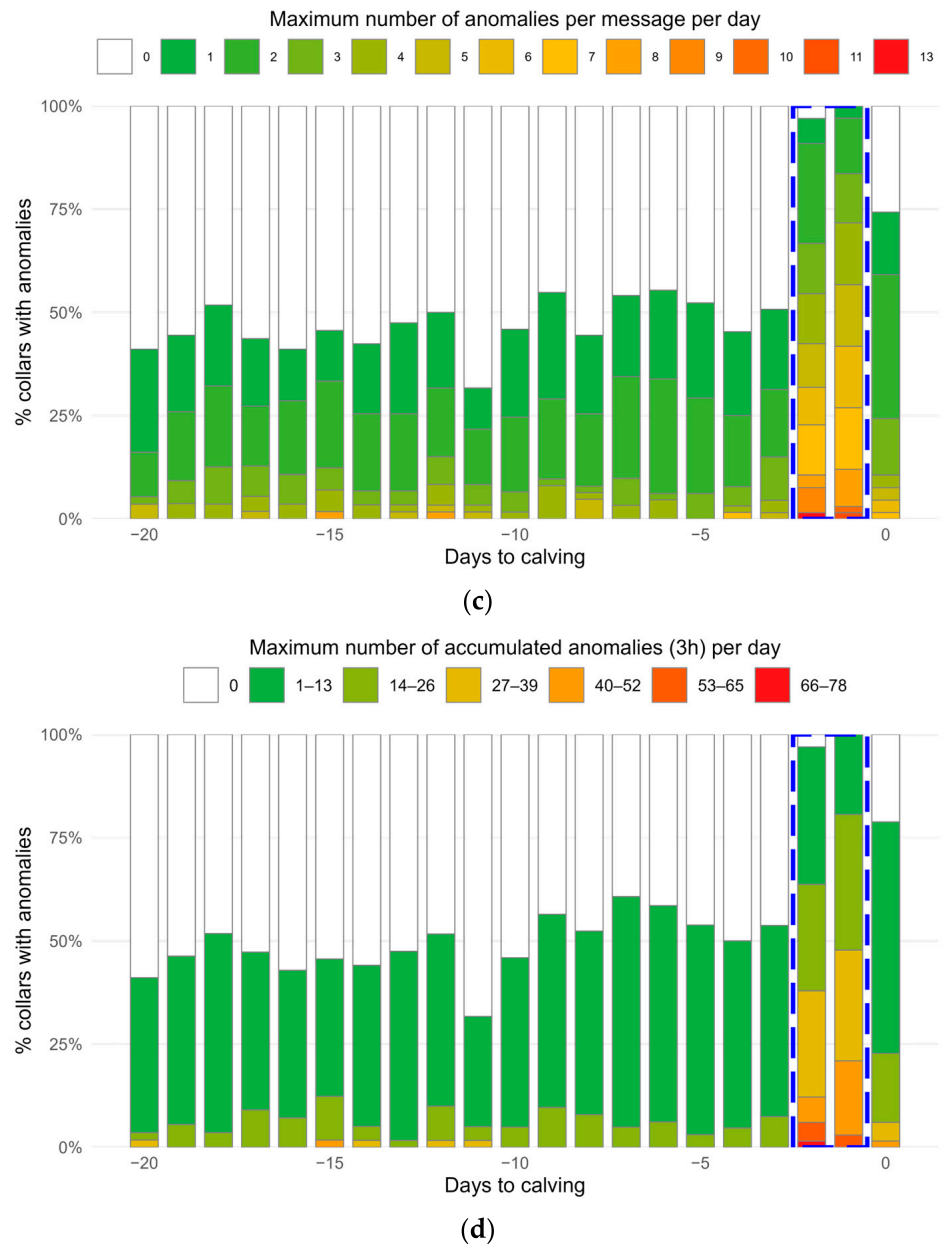
Temporal distribution of daily maximum anomaly counts during the 20 days before calving and the first day after calving: (**a**) anomalies at each 30 min timestamp calculated with a single collar; (**b**) anomalies accumulated over 3 h windows calculated with a single collar; (**c**) anomalies at each 30 min timestamp calculated with multiple collars; (**d**) anomalies accumulated over 3 h windows calculated with multiple collars. The calving window is indicated by a blue dashed line box.

The temporal distribution of alerts showed that behavioral anomalies were not evenly distributed across the 20-day evaluation period preceding calving (Figure 3a–d). In all scenarios, the proportion of collars generating alerts increased within the defined calving window.

When only individual indicators were evaluated at each 30 min timestamp, most cow-days showed no anomalies throughout the monitoring period (Figure 3a). Alerts based on one or two anomalous indicators occurred sporadically across the entire 20-day period, generally affecting a small proportion of collars. However, the proportion of collars with at least two anomalies increased during the final days before calving, reaching its highest value within the calving window. Alerts involving three simultaneous anomalies were rare outside the calving window but were present in 19% of collars on the day before calving.

A similar but more structured pattern was observed when individual-indicator anomalies were accumulated over a 3 h rolling window (Figure 3b). Low-level accumulated anomalies were still observed throughout the 20-day period, but the proportion of collars with accumulated alerts increased during the final days before calving. In addition, higher accumulated anomaly categories were more evident within the calving window than during the earlier prepartum period. This suggests that temporal accumulation helped distinguish repeated behavioral deviations close to calving from isolated anomalies occurring earlier in the monitoring period.

When the complete set of indicators was evaluated at each 30 min timestamp, alerts were more frequent across the entire 20-day period (Figure 3c). Low-level alerts, mainly involving one or two anomalous indicators, were observed on most days and affected a larger proportion of collars than in the individual-indicator scenario. However, the proportion of collars with alerts increased markedly during the calving window, where all collars showed at least one anomaly and higher anomaly categories became more frequent. Alerts involving three or more anomalies were relatively uncommon during the earlier prepartum period but became more prominent in the final days before calving.

The clearest temporal pattern was observed when the complete indicator set was combined with 3 h rolling accumulation (Figure 3d). In this scenario, low-level accumulated alerts were present throughout the 20-day period, reflecting the greater number of indicators included in the system. Nevertheless, the proportion of collars with alerts increased strongly during the calving window, reaching 100% on day −1. Importantly, higher accumulated anomaly categories were concentrated mainly during the calving window, whereas earlier days were dominated by low-level alerts. This pattern indicates that the accumulation of multiple anomalies over time strengthened the temporal association between alert severity and the calving period.

The temporal analysis showed that low-level alerts occurred throughout the prepartum period, especially when social and herd-relative indicators were included. In contrast, higher numbers of simultaneous or accumulated anomalies were concentrated around calving. This supports the rationale for using anomaly accumulation. While individual anomalies may reflect background behavioral variability, the occurrence of multiple anomalies within a short period was more closely associated with the calving window.

### 3.5. Anomaly Patterns Inside and Outside the Calving Window

**Figure 4:**
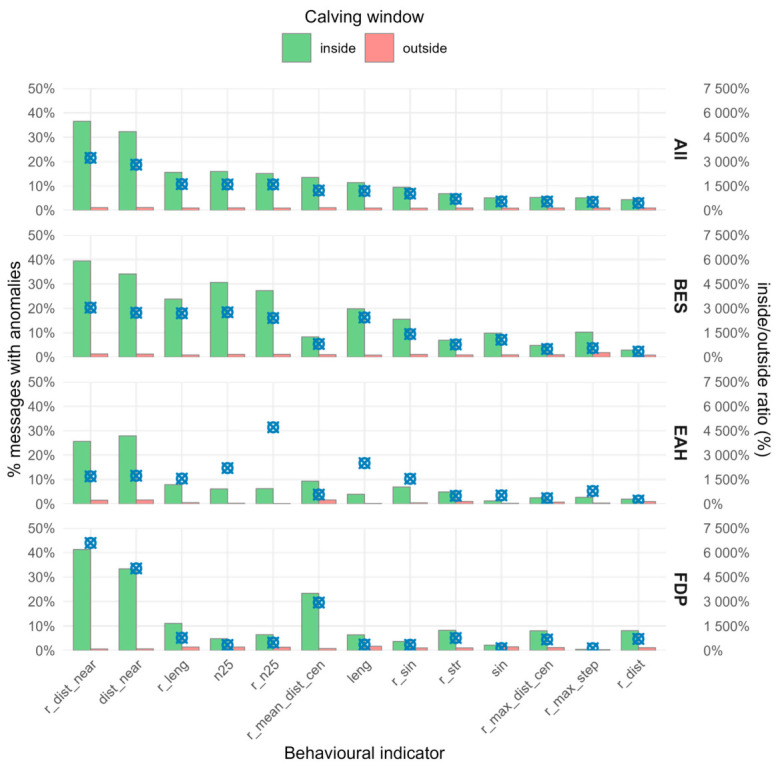
Proportion of collar messages (30 min time intervals) including anomalies inside and outside the calving window by behavioral indicator and farm (bars). Inside/outside the calving window anomaly ratio (blue symbol).

Figure 4 shows the proportion of anomaly detections for each behavioral indicator inside and outside the calving window, both overall and separately by farm. Across all farms, most indicators showed a higher proportion of anomalies inside the calving window than outside it. This contrast was particularly marked for indicators related to social spacing and relative movement, although the magnitude of the response varied across farms.

Overall, the indicators with the clearest contrast between inside and outside the calving window were relative distance to the nearest neighbor, distance to the nearest neighbor, relative trajectory length, number of segments exceeding 25 m, and relative number of segments exceeding 25 m. Within the calving window, anomalies in all these indicators occurred in more than 10% of the 30 min intervals, equivalent to at least 4.8 h over the 48 h period. For the relative distance to the nearest neighbor and the distance to the nearest neighbor, anomalies occurred in more than 30% of intervals, equivalent to over 14.4 h. They also showed high inside/outside ratios, with anomaly rates between 15 and 32 times higher inside than outside the calving window. This indicates that anomalies in these indicators were more concentrated around calving than during the previous non-calving days. Other indicators also showed higher anomaly rates inside the calving window, although with smaller contrasts. The relative distance between the start and the end of daily trajectories showed the smallest value for the inside/outside ratio, although anomalies for this indicator were 4.5 times more likely to occur inside the calving window.

Marked differences were observed among farms. ‘Belmont Station’ showed the highest anomaly rates for most indicators, with particularly strong responses for relative distance to the nearest neighbor and distance to the nearest neighbor. At this farm, anomalies inside the calving window were consistently higher than outside it across most indicators, indicating a clear temporal concentration of behavioral deviations around calving.

‘Fuente del Perro’ farm showed an intermediate pattern. The highest anomaly rates were observed for relative distance to the nearest neighbor, distance to the nearest neighbor, and relative mean distance to the centroid. The magnitude of the anomaly rates for these indicators was similar to that observed in BES. However, the anomaly rate for relative mean distance to herd centroid was higher than in BES. Meanwhile, the anomaly rate for locomotion-related indicators, such as relative trajectory length and the number of segments exceeding 25 m, was much lower.

‘El Ahijón’ farm showed the lowest anomaly rates overall. For most indicators, the proportion of anomalies was lower both inside and outside the calving window, and the inside/outside contrast was less pronounced than in the other farms. Interestingly, the relative number of segments exceeding 25 m showed the highest inside/outside ratio, but its overall anomaly rate was low, indicating that although anomalies in this indicator were relatively specific to the calving window, they occurred infrequently.

Figure 4 indicates that not all behavioral indicators contributed equally to calving detection. Indicators related to spatial separation from herd mates and relative movement showed the clearest concentration of anomalies inside the calving window, whereas other indicators contributed less consistently or showed weaker temporal specificity. Farm-specific results also indicate that the behavioral expression of calving-related anomalies was not uniform across sites, with BES showing the strongest signal, FDP an intermediate response, and EAH the weakest response.

## 4. Discussion

### 4.1. Study Approach

The present study developed and evaluated a near real-time calving detection system based on the accumulation of behavioral anomalies derived exclusively from GNSS tracking data. The main contribution of this approach is that it provides an interpretable alternative to supervised machine learning models when the exact timing of calving is not known. In extensive grazing systems, calving is often confirmed retrospectively, when the cow is first observed with the calf, rather than at the precise moment of birth. Under these weakly labeled conditions, training a supervised classifier would require assigning artificial calving times and could introduce false precision. The anomaly accumulation framework used here avoids this limitation by identifying deviations from each cow’s recent behavior and evaluating whether these deviations concentrate within a biologically plausible calving window.

The maximum F1-score obtained in the present study was 0.58 when the complete indicator set was used, compared with 0.41 when only individual indicators were considered. These values are lower than the highest performance reported in previous studies, mostly based on multi-sensor approaches [33]. However, the magnitude and interpretation of the difference depend strongly on the validation framework.

Chang et al. [24] analyzed nine cows with eutocic calvings and obtained 88.5% sensitivity, 100% precision, and 100% specificity using GNSS, accelerometer-derived rumination, and weather data. Although the F1-score was not reported, these precision and sensitivity values would correspond to an F1-score above 0.9. Their evaluation was also conducted on a cow-day basis, but involved a single farm, a seven-day evaluation period, and the exclusion of four cows with dystocia or adverse calving outcomes.

Fogarty et al. [23] evaluated their GNSS–accelerometer–weather model using 11 independently validated lambing events with birth times known at approximately hourly resolution. Depending on the alert rule, only 27% or 55% of events were detected within ±3 h of birth without prior false-positive alerts. Detection increased to 91% and 82%, respectively, when animals with earlier false alerts but a subsequent correct alert were considered successfully detected; however, these configurations generated 64 and 22 prior false-positive alerts. Thus, the reported percentages represent event-level sensitivity under different tolerance levels for early false alerts rather than F1-scores directly comparable with those obtained in the present study. Their findings also highlighted the importance of social-distance variables and temporal decision rules for detecting parturition.

More recently, Wang et al. [25] correctly classified calving in 12 of 15 cows using a GNSS–accelerometer autoencoder and Random Forest framework, while two calvings were missed and one was located approximately 47 h from the recorded event. Moreover, anomalies within ±24 h of the recorded event were labeled as calving-related, and predictions separated by up to 24 h were clustered as a single event. Their 80% event-level detection rate was therefore obtained using a different temporal definition and evaluation unit.

These comparisons confirm that the performance of the present GNSS-only framework was lower than the best result reported by Chang et al. [24] and lower than the event-detection rates obtained under some configurations of Fogarty et al. [23] and Wang et al. [25]. However, these numerical differences should not be interpreted as a direct ranking of algorithms, as the studies differed in species, sample size, sensor inputs, precision of event labels, evaluation horizon, treatment of repeated or early alerts, and definitions of true- and false-positive detections. The present study included 68 valid calving events across three farms and applied a conservative cow-day evaluation under weakly labeled commercial conditions. Its contribution therefore lies not in matching the maximum predictive performance obtained in smaller or more tightly controlled multi-sensor studies, but in evaluating the performance and operational trade-offs of a transparent GNSS-only framework under heterogeneous real-world deployment conditions.

Multi-sensor studies nevertheless demonstrate the potential benefit of integrating complementary behavioral information. However, such systems also involve additional hardware, more frequent and heterogeneous data streams, and more complex procedures for transmission, synchronization, processing, and model training. In contrast, the present approach derives all behavioral indicators from commercial GNSS collar data. It should not therefore be expected to outperform systems that incorporate accelerometry, weather, or weight information, but it may provide a favorable compromise among predictive performance, interpretability, and operational feasibility in extensive beef systems, where connectivity, battery life, labor availability, and device robustness are important constraints.

A further practical feature of the proposed framework is that the same analytical procedure was applied to data obtained from different collar models and communication architectures. Harmonizing trajectories to a common temporal resolution and defining anomalies relative to each cow’s recent behavior may facilitate transfer across GNSS platforms compared with supervised models trained on a single device-specific data distribution. Nevertheless, complete device independence should not be assumed, because GNSS accuracy, missing-data patterns, and sensor configuration may still influence some behavioral indicators. Validation and, where necessary, recalibration should therefore accompany transfer to new collar platforms. Finally, the scale and diversity of the dataset represent an important contribution of this study. Previous calving-detection studies have generally involved a small number of parturition events or a single experimental setting. In contrast, the present analysis included 68 valid calvings recorded across three grazing systems in Spain and Australia, encompassing contrasting collar technologies, monitoring strategies, herd structures, and management conditions. This multi-farm and multi-country design enabled the anomaly-based framework to be evaluated under a broader range of practical conditions, while also revealing the context dependence of its performance.

### 4.2. Effect of Anomaly Thresholds and Temporal Accumulation

The results showed that the number of anomalies required to trigger a calving alert strongly influenced detection performance. As expected, lower thresholds increased recall but produced more false-positive cow-days, whereas higher thresholds improved precision at the cost of missing more calving events. This trade-off is central to practical deployment because the preferred balance between sensitivity and false alerts will depend on farm context, calving risk, and labor availability.

A key point is that thresholds must be interpreted relative to the maximum number of possible anomalies in each scenario. In the individual-indicator scenario, only three anomalies could occur at a single 30 min timestamp, whereas up to 18 anomalies could accumulate over 3 h. In the complete indicator set, the corresponding maxima were 13 and 78 anomalies. Therefore, a given numerical threshold does not have the same meaning across scenarios.

The use of a 3 h accumulation period improved the stability of the alert system by reducing the influence of isolated behavioral deviations. Individual anomalies may arise from normal grazing routines, short-term environmental effects, interactions with other animals, management events, or GNSS noise, whereas repeated anomalies over a short period are more likely to reflect a sustained behavioral disruption. However, temporal accumulation also introduces an operational trade-off. Requiring anomalies to accumulate before an alert is triggered may delay warning generation relative to a 30 min rule, thereby reducing the lead time available for farmer intervention. The magnitude of this delay depends on the selected anomaly threshold and on how rapidly anomalies accumulate, and it is therefore not necessarily equal to the full 3 h window.

This trade-off is consistent with previous parturition-monitoring studies showing that temporal decision rules can improve alert reliability but may alter sensitivity and detection timing [24,25]. In the present study, temporal accumulation served a similar purpose but within a transparent rule-based framework rather than a supervised classification model. Accordingly, the accumulation period should be selected by balancing alert stability and false-positive reduction against the need for timely pre-calving warning.

### 4.3. Temporal Concentration of Alerts Before Calving

The temporal distribution of alerts supported the biological relevance of the anomaly-based approach. Low-level alerts occurred throughout the 20-day evaluation period, particularly when social and herd-relative indicators were included. In contrast, higher anomaly counts were concentrated in the final two days before confirmed calving, especially when anomalies were accumulated over 3 h. This distinction indicates that isolated behavioral deviations are common under grazing conditions, whereas the concurrent or repeated occurrence of several anomalies provides a more specific signal of an impending calving event.

This pattern could support a tiered alert system for practical implementation. For example, isolated or low-level anomalies could be retained as background information without prompting immediate intervention, whereas intermediate anomaly counts could generate a low-priority warning or place the cow on a watch list. Only high anomaly counts or sustained accumulation over consecutive intervals would trigger a high-priority alert requiring inspection. Such a structure could reduce unnecessary interventions while preserving information on the progressive development of prepartum behavioral changes. The thresholds defining each alert level could be adjusted according to herd value, labor availability, paddock accessibility, weather conditions, and the relative costs of missed calvings and false alerts. A complementary strategy would be to adopt a two-stage framework similar to that proposed by Wang et al. [25], in which an initial anomaly detection stage identifies unusual behavioral episodes and a second classification stage distinguishes calving-related anomalies from deviations caused by other events.

The observed pattern agrees with previous GNSS-based studies showing that calving-related changes become more evident during the final day or two before parturition. García-García et al. [17] reported that social indicators, especially distance to the herd centroid and distance to the nearest peer, were more useful than individual movement indicators for detecting calving day, with cows showing isolation behavior from approximately 24 h before calving. The present results extend this idea by showing that near real-time anomaly accumulation can translate these behavioral changes into operational alerts.

Further improvement could involve dynamically updated behavioral baselines. The current framework already compares each observation with the cow’s recent behavior, but future implementations could adapt the baseline and anomaly thresholds according to season, paddock, forage conditions, management events, and recent false-alert patterns. This would allow the system to respond to gradual changes in normal behavior without interpreting them repeatedly as calving-related anomalies.

The evaluation period used in this study should also be considered when interpreting performance metrics. Metrics were calculated over the 20 days preceding recorded calving, rather than across the entire monitoring period. This period represents a practical horizon for calving surveillance, but it restricts the number of non-calving days compared with an evaluation over the full dataset. Consequently, false-positive occurrence and precision may differ during continuous year-round implementation.

### 4.4. Biological Interpretation of Anomalies

The indicators with the clearest contrast between inside and outside the calving window were mainly related to social separation and relative movement. These indicators are consistent with two complementary behavioral mechanisms: temporary isolation from herd mates before calving and reduced locomotion relative to the rest of the group around calving. However, GNSS-derived separation should not be interpreted as direct evidence of voluntary social withdrawal alone, because the observed distances may also be influenced by the spatial distribution of pasture resources and paddock infrastructure.

The importance of social separation is well supported by previous ethological work. Flörcke and Grandin [15] reported that beef cows showed separation behavior before parturition, with many cows moving more than 100 m from the herd or feeding area before calving. This behavior is generally interpreted as a temporary social withdrawal that may facilitate cow-calf bonding and reduce disturbance around birth. In natural environments, it may also help minimize predation risk for the newborn. The present results support that GNSS-derived metrics can capture this temporary isolation as a measurable anomaly. Nevertheless, the spatial expression of this behavior is likely to depend on the structure of the paddock. Water points, shade, forage patches, topography, and fencing layouts may concentrate or disperse animals and can therefore produce apparent segregation even in the absence of active isolation. Conversely, these same features may facilitate or constrain a cow’s ability to separate from the group.

The relevance of social-distance indicators also agrees with previous sensor-based studies. Fogarty et al. [23] identified mean and minimum distance to peers among the most important features for detecting lambing under grazing conditions, even though isolation behavior is more subtle in sheep. García-García et al. [17] found that distance to herd centroid and neighbors were key indicators for identifying calving in beef cows. Chang et al. [24] reported that GNSS-derived information contributed strongly to the best-performing multi-sensor calving detection models. Wang et al. [25] also reported increased deviations in spatial behavior prior to calving. Taken together, these studies indicate that spatial divergence from groupmates is a recurrent signal of parturition.

The locomotion-related indicators with increased anomalies within the calving window reflect a reduction in cow movement relative to herd mates. As calving approaches, cows may reduce exploration, remain closer to the selected birth site, or deviate from the normal grazing pattern followed by the rest of the group. This transition has been reported in accelerometer-based work as a reduction in walking distance or step counts [25], and in GNSS-based studies as shorter trajectories and smaller home ranges [17,24]. Moreover, the first hours after calving are characterized by intense maternal behavior, including licking and suckling, which often comes at the expense of cow feeding behavior [34], resulting in reduced spatial movement. Relative indicators are particularly useful in this context because they account for the behavior of groupmates exposed to the same paddock, weather, and management conditions. Thus, they capture not only whether a cow moved less, but whether she moved differently from the group.

Some indicators showed weaker or less specific patterns. Relative straightness, sinuosity, relative maximum distance to herd centroid, relative maximum segment length, and relative distance contributed less consistently to calving detection. These metrics may be more sensitive to paddock characteristics (e.g., terrain, fences, water points, or shade distribution) and routine grazing movements than to calving itself. For example, relative distance was intended to capture changes in a cow’s normal daily route when she separates from the herd before calving. However, the magnitude and detectability of this change are likely to depend on paddock size and configuration. In small paddocks, cows may have limited opportunity to alter their route relative to groupmates. Similarly, the location of water troughs or shade areas may shape the routes followed by both calving and non-calving animals. A cow seeking isolation may move towards less crowded areas of the paddock, but if these areas are close to key resources, the resulting change in trajectory may be small or inconsistent.

These findings have direct implications for model optimization. Indicators showing consistent contrasts across farms, particularly nearest-neighbor distance and relative movement metrics, should form the core of the alert system. In contrast, indicators that are strongly affected by paddock structure or that contribute little additional information could be assigned lower weights, excluded from high-priority alert rules, or retained only when their local performance has been confirmed. A tiered variable-selection strategy could therefore distinguish core indicators, which contribute systematically to alert generation, from supplementary indicators, which provide contextual support but are not sufficient to trigger an alert alone.

This does not necessarily mean that weaker indicators are biologically irrelevant, but they may be less reliable as standalone indicators for alert generation. Previous studies [23,25] have reported similar findings in their feature-importance analyses, showing that a subset of spatial and activity variables carries most of the predictive power, while others contribute little once the core mechanisms are accounted for.

### 4.5. Farm-Specific Differences

Anomaly detection patterns differed among farms, with BES generally showing the strongest signal, FDP an intermediate response, and EAH the weakest and least persistent response. These differences indicate that the performance of a GNSS-based calving detection system is determined not only by the algorithm but also by farm and paddock conditions, herd management, and the extent to which collared animals represent the behavior and social structure of the herd.

Differences among collar technologies may have also contributed to the farm-specific patterns observed. Although variation in data completeness and temporal resolution was reduced through data-quality filtering and resampling to 30 min intervals, differences in GNSS positional accuracy could not be fully removed. Indicators based on fine-scale trajectory geometry, such as sinuosity and the number of segments exceeding 25 m, may be particularly sensitive to location error. For some of these indicators, the contrast in anomaly frequency between inside and outside the calving window was greater at BES than at the Spanish farms. This pattern may partly reflect differences in positional accuracy between the i-gotU and Digitanimal devices. However, the collar model was fully confounded with farm, management conditions, and animal population, and its independent effect could not therefore be quantified. This explanation should consequently be interpreted with caution.

The proportion of animals fitted with GNSS collars also appears to be an important factor. This is particularly relevant for social and herd-relative indicators, because these metrics are calculated only from monitored animals. At BES, all animals were collared and managed as a single group within the same paddock. Therefore, indicators such as distance to the nearest neighbor or distance to the herd centroid were likely to represent the true social environment of each cow with relatively high accuracy. In contrast, collar coverage was lower at the Spanish farms, particularly at EAH, where only 21% of cows were collared at a given time. Under these conditions, the nearest monitored neighbor may not be the true nearest neighbor. A cow may be physically close to an uncollared groupmate but appear isolated in the dataset, which could increase the background variability of social-distance indicators and reduce their specificity for calving. This partly explains why EAH showed lower anomaly rates for social indicators. In addition, collars at EAH were allocated preferentially to cows considered likely to calve in the near term. Although this deployment reflects a realistic commercial strategy under limited collar availability, the monitored cows were not randomly selected, which may have introduced selection bias and limits direct inference to the entire calving population.

Incomplete collar coverage may also affect relative indicators. Herd-relative metrics assume that the monitored animals provide a reliable description of the behavior of the group occupying the same paddock. If only a fraction of the herd is collared, the calculated group average may not fully reflect the actual movement pattern of the whole group. This limitation may become more relevant when farmers divide the herd into different paddocks according to management needs. In those cases, the proportion of collared animals within a given paddock group may be lower than the overall collaring rate of the farm, reducing the reliability of herd-relative indicators for that group.

These results do not imply that high or complete collar coverage is required for practical implementation. Under low-coverage conditions, the alert system could place greater emphasis on individual movement indicators, which do not depend on the proportion of monitored groupmates, while assigning lower weight to social and herd-relative metrics. Social indicators could also be used only when a minimum number or proportion of animals within the same paddock is being monitored, or their contribution could be adjusted according to the local collar coverage.

Management and paddock conditions at the time of calving probably also contributed to between-farm differences. The detectability of reduced locomotion around calving depends on the contrast between the behavior of the calving cow and the normal movement pattern of the herd. For example, when forage availability is low or spatially heterogeneous, non-calving animals may need to travel longer distances each day to find food. In that context, a cow that reduces movement or remains near a selected calving site may differ more clearly from her groupmates, making relative trajectory length or relative number of segments exceeding 25 m more informative. Conversely, if forage, water, shade or supplementary feed are concentrated in specific areas, the whole herd may show more restricted or clustered movement [35], reducing the contrast between calving and non-calving animals. Paddock configuration on each farm may also influence the differences in some indicators. If water points, shade or feeding areas determine the main movement routes of the herd, a cow seeking isolation may move towards less crowded areas without necessarily producing a large change in net displacement or trajectory shape. Similarly, supplementation practices may also have affected the spatial behavior of the herd and the discriminatory value of some indicators.

Parity may also have contributed to the higher anomaly rates observed at BES. A larger proportion of animals at this site were primiparous, and primiparous cows may express stronger or more detectable isolation behavior around calving than multiparous cows [36]. If primiparous animals separate more clearly from the herd or show more pronounced changes in movement before parturition, this could amplify the anomaly signal, particularly for indicators related to social distance and relative locomotion.

Overall, the farm-specific patterns observed in this study suggest that GNSS-based calving detection is context-dependent. Differences in collar technology, particularly in positional accuracy, may affect indicators based on fine-scale movement geometry. Complete or high collar coverage may improve the reliability of social and herd-relative indicators, whereas partial collar coverage can introduce uncertainty into the apparent social position of each cow. In addition, forage availability, supplementation, resource distribution, paddock configuration and parity may all influence the contrast between normal grazing behavior and calving-related behavioral deviations. These findings support the need for external validation across farms and suggest that future implementations may benefit from farm-specific calibration or adaptive thresholds that account for the varying conditions found on different farms [5].

### 4.6. Practical Implications and Future Research

From an applied perspective, the flexibility of the anomaly threshold approach is one of its main strengths. Different farms may require different balances between recall and precision. In remote or labor-limited systems, producers may prefer stricter thresholds that generate fewer alerts, even if some calving events are missed. Conversely, in more intensively supervised herds, lower thresholds may be acceptable because staff can respond to more alerts and the priority may be to avoid missing calving events. This means that a single universal threshold is unlikely to be optimal for all farms.

Rather than prescribing fixed threshold values for broad farm categories, which would not be supported by the present datasets, implementation should involve local calibration using historical farm data. A practical procedure would be to evaluate several candidate thresholds, quantify the associated precision, recall, and daily alert burden, and then select the operating point that best reflects the farm-specific cost of missed calvings relative to unnecessary inspections. In remote low-labor systems, this would generally favor thresholds with higher precision and lower alert frequency, whereas high-value or closely supervised herds may accept lower precision in exchange for higher recall.

The threshold-response curves reported in this study can therefore be used as a reference for the direction of these trade-offs, but not as universal engineering specifications. Nonetheless, the choice of a ground indicator in known behavioral mechanisms provides a transparent rationale for model configuration and facilitates the communication of alerts to end users.

Configurable or adaptive thresholds may therefore be a promising route for implementation. A practical system could allow users to select a more sensitive or more conservative alert setting depending on calving risk, herd value, labor availability, paddock accessibility, or weather conditions. Future work could also evaluate farm-specific thresholds, dynamic baselines, or adaptive algorithms that account for paddock, season, forage availability, supplementation, and other practices affecting cow spatial behavior. The temporal and statistical parameters used in the present study should likewise be regarded as biologically informed operating choices rather than universally optimal settings. Shorter baseline windows may increase responsiveness but also make the system more sensitive to normal circadian variation, whereas longer windows may provide more stable baselines at the cost of smoothing short-term prepartum changes. Similarly, the percentile used to define indicator-specific anomalies, the duration of the accumulation window, and the anomaly count threshold may alter the balance between sensitivity, false-alert frequency, and alert latency. Future implementations could therefore fine-tune these parameters through sensitivity analyses or farm-specific calibration according to collar resolution, management conditions, and the operational consequences of missed calvings and false alerts.

Several limitations of the present study should also be acknowledged. First, exact calving times were not available, and validation was based on calving windows rather than precise birth timestamps. This reflects the reality of extensive grazing systems but limits the ability to evaluate the exact temporal accuracy of alerts. Second, the analysis was restricted to the 20 days preceding calving, which may affect the interpretation of false-positive rates compared with continuous deployment. Third, farm-specific differences indicate that the algorithm may require local calibration before operational use. Finally, the system was evaluated retrospectively.

Future validation should therefore be conducted prospectively under commercial conditions, with alerts generated and transmitted in near real time. Such trials should include farms representing contrasting management systems, collar-coverage levels, paddock structures, and labor availability. Exact or narrower calving times should be established whenever possible through direct observation or the use of complementary sensors, allowing alert lead time, duration, persistence, and false-alert frequency to be quantified more precisely.

Prospective trials should also record the farmer’s response to each alert, including whether the animal was inspected, the time required to reach it, whether intervention was considered necessary, and whether the alert contributed to the detection of dystocia, calf loss, or other calving-related problems. These data would make it possible to evaluate not only algorithmic performance but also alert usefulness, workload, response compliance, and alert fatigue. This would provide a more realistic assessment of field performance and practical value before commercial deployment.

## 5. Conclusions

This study shows that near real-time calving alerts can be generated for grazing cows using an anomaly-based framework based exclusively on GNSS tracking data. By combining individual, social and herd-relative behavioral indicators with short-term temporal accumulation, the system detected biologically meaningful peripartum deviations, mainly related to increased spatial separation from groupmates and reduced movement relative to the group. The use of anomaly accumulation was particularly relevant, as isolated anomalies were common throughout the prepartum period, whereas the concentration of multiple anomalies within short time windows was more closely associated with the calving window.

The proposed approach provides a transparent and flexible alternative to supervised machine learning models in situations where the exact timing of calving is unknown. Its reliance on commercially available GNSS collars, without additional sensors, supports its potential use in extensive grazing systems where simplicity, cost-effectiveness and ease of deployment are essential. However, the results also indicate that performance is context-dependent, being influenced by collar coverage, herd management, paddock conditions and farm-specific behavioral patterns.

Future research should investigate alternative time windows for calculating indicators and for rolling accumulation, which may further improve responsiveness and noise filtering. Testing weighted anomaly schemes, where indicators with stronger discriminative value contribute more heavily to alert generation, could also refine performance.

## Figures and Tables

**Table 1:** Main characteristics of cattle monitoring at the three study locations.

Farm Name	Country	Herd Size	Monitored Cows	Monitoring Period Length	Collar Type	Sampling Interval
‘Fuente del Perro’	Spain	75	57	9 months	Digitanimal (SigFox)	30 min
‘El Ahijón’	Spain	95	42	8 months	Digitanimal (GSM)	30 min
‘Belmont Station’	Australia	50	50	3 months	i-gotU (on-board storage)	5 min

## Data Availability

The data presented in this study are available on request from the corresponding author due to privacy concerns.

## Abbreviations

The following abbreviations are used in this manuscript:

BES	Belmont Station
EAH	El Ahijón
FDP	Fuente del Perro
GNSS	Global Navigation Satellite System
GPS	Global Positioning System
GSM	Global System for Mobile Communications
SVM	Support Vector Machine
